# Predicting special forces dropout via explainable machine learning

**DOI:** 10.1002/ejsc.12162

**Published:** 2024-09-24

**Authors:** Rik Huijzer, Peter de Jonge, Frank J. Blaauw, Maurits Baatenburg de Jong, Age de Wit, Ruud J. R. Den Hartigh

**Affiliations:** ^1^ Faculty of Behavioural and Social Sciences Department of Developmental Psychology University of Groningen Groningen the Netherlands; ^2^ Research and Innovation Researchable BV Assen the Netherlands; ^3^ Ministry of Defence Den Haag the Netherlands

**Keywords:** assessment, military selection, performance, performance prediction, SIRUS model

## Abstract

Selecting the right individuals for a sports team, organization, or military unit has a large influence on the achievements of the organization. However, the approaches commonly used for selection are either not reporting predictive performance or not explainable (i.e., black box models). In the present study, we introduce a novel approach to selection research, using various machine learning models. We examined 274 special forces recruits, of whom 196 dropped out, who performed a set of physical and psychological tests. On this data, we compared four machine learning models on their predictive performance, explainability, and stability. We found that a stable rule‐based (SIRUS) model was most suitable for classifying dropouts from the special forces selection program. With an averaged area under the curve score of 0.70, this model had good predictive performance, while remaining explainable and stable. Furthermore, we found that both physical and psychological variables were related to dropout. More specifically, a higher score on the 2800 m time, need for connectedness, and skin folds was most strongly associated with dropping out. We discuss how researchers and practitioners can benefit from these insights in sport and performance contexts.

## Highlights

- In high‐stakes contexts such as elite sports and the military, selecting the right individuals has a large influence on individual and organizational achievements.
- We compare four machine learning models, with various levels of explainability, on a dataset of 274 Dutch special forces recruits.
- A fully explainable SIRUS model could achieve an average area under the curve score of 0.70 for predicting dropouts from the program.
- Both physical and psychological variables were related to dropout, especially the 2800 m time, need for connectedness, and skin folds.

## 1 INTRODUCTION

The achievements of sports clubs, organizations, and military units are largely determined by the performance of the individuals in the organization. As a consequence, there is an ever increasing pressure to select the right individuals, that is, individuals who will perform successfully in the future (e.g., Den Hartigh, Niessen, et al., 2018). Historically, military selection has been an important breeding ground for research into selection in psychology and sports. For example, widely used instruments such as intelligence tests (Terman, 1918), personality inventories (Ellis & Conrad, 1948), and leadership measures (Fleishman, 1953) were first established and validated in military contexts. In the present study, we aimed to advance the field of selection further by applying machine learning models for the selection of elite soldiers. In doing so, we set out to investigate the predictive performance, explainability, and stability of statistical models based on relevant physical and psychological predictors. Here, predictive performance means the estimated ability of the model to predict future behaviors, explainability means how easy it is to understand the model and why certain predictions were made, and stability means the ability of the model to produce similar conclusions for small changes to the data (Yu, 2013).

### 1.1 Selection in high‐stakes military contexts

Within the military, the special forces are considered elite. Special forces operators need to be able to perform their tasks under difficult circumstances, such as continuous threat, extreme temperatures, isolation, and high task complexity, while being involved in politically sensitive situations (Picano et al., 2002). Similar to elite sports, this requires extraordinary physical and mental capabilities (Vaara et al., 2022). Special forces selection courses worldwide simulate these circumstances in, what some countries call, hell weeks. During these selection weeks, recruits typically complete exercises and tasks for a large part of the day while being sleep‐deprived. Several studies have been conducted in the past decades to predict success versus dropout in such selection programs of the special forces. For example, a study among 800 candidates found that both physical and psychological measures, such as grit and pull‐ups, were significantly correlated with graduation (Farina et al., 2019). The relevance of physical and psychological factors were also found in other high‐stakes military contexts. For instance, studies on 12,924 military pilots, 115 reconnaissance marines, and 57 counter terrorism intervention unit recruits found that various physical and psychological measures were associated with graduation (King et al., 2013; Saxon et al., 2020; Tedeholm et al., 2021). Furthermore, a large‐scale study on 1138 United States (U.S.) special forces candidates found that psychological hardiness was significantly correlated with graduation (Bartone et al., 2008). Taken together, a multidisciplinary approach, including both physical and psychological measures, is likely to perform best on the complex task of predicting success (Williams & Reilly, 2000).

An important note about previous research is that many studies report only model explanations, that is, the studies fit a statistical model to the data and report the fitted parameters. Interestingly, this approach is also a common practice in the field of sport science. However, the outcomes produced by such models may have little ability to predict future behaviors because of overfitting (Hofman et al., 2021; Jauhiainen et al., 2022; Yarkoni & Westfall, 2017). Also, many studies only report the results from one statistical model, such as a simple regression or the *t*‐test, which largely ignores the statistical (and computational) progress made since then. Applying more recent analytic techniques, such as model evaluation via cross‐validation, could therefore improve research into the selection procedures (e.g., Abt et al., 2022).

### 1.2 Statistical models from machine learning

Recent analytic advances can be found in the domain of machine learning, which can generally be described as computer systems that learn and adapt without following specific instructions. One example is computer vision, which contains models that can learn from visual data to automatically detect and classify sport‐specific movements. In general, the field invented and re‐discovered a plethora of statistical models, many of which are promising because the models are distribution‐free and are able to find complex relationships in data. The distribution‐free property is relevant for selection because psychometric variables are usually normally distributed, while performance variables in elite performers often are not (e.g., Den Hartigh, Hill, et al., 2018; O’boyle Jr & Aguinis, 2012). Furthermore, finding complex relationships could provide new insights into the underlying processes when sufficient data are available. As an example, Jauhiainen et al. (2022) used a complex data set, containing 3‐dimensional motion and physical data, to predict injuries in 791 female elite handball and soccer players. More generally, the commonly applied random forest algorithms have been very performant in different settings, especially when the number of variables is large or larger than the number of observations (Biau & Scornet, 2016).

However, machine learning is no panacea. A disadvantage of many machine learning applications in sports and the selection of military personnel is that the models are too complex to understand. Often, the complex models are then converted to a simplified form to make them interpretable, for example, by using SHAP (SHapley Additive exPlanations; for details, see Molnar, 2022). Although the purpose of SHAP is to increase transparency and explainability of machine learning models, it loses information during the conversion from the complex model to the simplified representation. In other words, the simplified representation is not the same as the model that will be used for decision‐making. This is problematic for researchers and practitioners because the simplification could hide issues related to safety, fairness (e.g., biases), and reliability (Barredo Arrieta et al., 2020; Doshi‐Velez & Kim, 2017). This is especially important in the context of selection, where wrong decisions can have a lasting impact on the individual.

Apart from predictive performance and explainability, the stability of models is also an important aspect. A stable model is defined as a model which leads to similar conclusions for small changes to data (Yu, 2013). An example of an unstable model could be a model which selects personality and sprint times to predict dropout in this year's cohort but selects other variables for next year's cohort. In the context of selection, this variation in the prediction model is problematic. Unstable models can cause various operational problems such as being deemed less trustworthy and requiring constant changes to the selection procedure (Yu, 2013).

### 1.3 Current study

The purpose of the current study was to determine how well we could predict dropout of special forces recruits while retaining model explainability and stability. We used a regularized linear model as a baseline. This model is close to the linear models that are typically used for decision‐making in sport and psychology research. Next, we used three machine learning models, namely a decision tree, a state‐of‐the‐art random forest, and a state‐of‐the‐art explainable rule‐based model. We specifically investigated how the four models compared on their predictive performance, explainability, and stability. We compared the models on their predictive performance via average area under the curve (AUC), on their explainability by comparing model interpretation techniques (e.g., linear model coefficients vs. SHAP), and stability by comparing the differences between the algorithms used.

## 2 MATERIALS AND METHODS

### 2.1 Participants

We gathered data of 311 participants aged between 20 and 39 (M*a*ge = 26.5, SD*a*ge 3.8), who were exclusively Dutch males and all part of the selection of the Special Forces of the Royal Netherlands Army. Active consent was obtained from all participants and the procedure was approved by the ethical review board of the faculty (code: PSY‐1920‐S‐0512). Data preprocessing, which included the removal of participants for which some data was missing, resulted in a dataset of 274 participants. Of these participants, 196 dropped out and 78 graduated. More information could not be provided due to security reasons.

### 2.2 Design and procedure

Participation occurred via a platform specifically built for the research project (Your Special Forces). The data collection was organized by researchers of the university at the training camp and was facilitated by the staff of the Special Forces unit. Physical assessments occurred on the first day of the first week. Also in the first week of the training, participants completed the psychological assessments using tablets in a large room which was set up like a traditional classroom. Once participants entered the room for the psychological assessment, they were informed about the consent procedure, study goal, and that participation would not affect their graduation chances. For three to 4 days, the participants spent roughly 1 hour per day on filling out the questionnaires, which were all in Dutch.

### 2.3 Measures

The study contained both physical and psychological measures. The physical fitness of the recruits was measured using a test battery designed to assess relevant physiological and physical characteristics that are considered to be important in military training courses (e.g., Haff & Triplett, 2015). All tests were taken in a predetermined order. First, body composition was determined by measuring length, weight, and the 4‐Site Skinfold (Durnin & Womersley, 1974). Then, a standardized warming up was conducted after which the recruits started in the test circuit. Lower body power was measured with a broad jump, and the best of three attempts was noted in centimeters. Next, speed and agility were tested using the Pro Agility test conducted twice with 30 s rest in between and both sprint times were summed. The agility test was followed by maximal grip strength of both hands with one attempt per hand using a Grip dynamometer. After this test, maximal strength of the lower body push and pull and upper body push‐kinetic chain was measured with a three repetition max (RM) protocol using the hex‐bar deadlift and bench press exercise. Strength endurance of the upper body pull‐chain was measured with pull‐ups: Recruits had 1 minute to complete as many pull‐ups as possible. The penultimate test was designed to determine the anaerobic capacity of the recruits using a 60‐m sprint. It measured the time it took to sprint from one place to a place 5 m away and back (10 m), then 10 m away and back (20 m), and finally to a place 15 m away and back (30 m). Also here, the test was conducted twice with 30 s in between. After the 60‐m sprint, the recruits had exactly 10 min to recover and prepare for the aerobic endurance test, a timed 2800‐m run. The recruits were instructed to complete 8 rounds on a 350‐m concrete track as fast as possible.

Regarding the psychological measures, the first day included the informed consent and questionnaires about resilience, goal commitment, and self‐efficacy. The resilience questionnaire assessed the ability to recover or bounce back from stress via the Brief Resilience Scale (Smith et al., 2008). For example, one of the six items was “I tend to bounce back quickly after hard times”. Next, goal commitment was measured via six items such as “I am strongly committed to pursuing my goals” (see Van Yperen, 2009). The next questionnaire measured self‐efficacy (Bandura, 2006) with 14 items such as “How confident are you in your ability to remain calm in difficult situations?”.

The second day consisted of two cognitive ability tests (Condon & Revelle, 2014). The first test contained 11 matrix reasoning items and the second test contained 24 three‐dimensional rotation items. The participants were allowed to take 15 and 30 min, respectively, to finish both tests. On the third day, three questionnaires were answered. The first questionnaire was a combination of five short questionnaires, namely Mindsets (Dweck, 2000), psychological need strength (Van Yperen et al., 2014), Motivation Type (Pelletier et al., 2013), and Approach‐Avoidance Temperament (Elliot & Thrash, 2010). The second measured mental toughness via the MTQ48 (Clough et al., 2002). This questionnaire contains four key components, namely Control, Commitment, Challenge, and Confidence. The third questionnaire measured Coping (Lazarus & Folkman, 1984). This questionnaire measured emotion‐focused versus problem‐focused coping in response to stressful events. For example, “I try to forget the whole thing by focusing on other things” which is an example of an emotion‐focused strategy. After this, the participants filled in the Dutch version of the NEO‐PI‐3 personality questionnaire, which measures the big five dimensions: Neuroticism, Extraversion, Openness, Agreeableness, and Conscientiousness (Hoekstra & De Fruyt, 2014).

## 3 ANALYSES

In order to find the best performing model, we compared four different models via MLJ.jl (Blaom et al., 2020). We calculated the models' scores on the Area Under the receiver operating characteristics Curve (AUC). The AUC is a metric that indicates how well a model predicts a binary outcome, dropout versus graduation in our case. The AUC takes into account that the threshold of the model can be chosen freely. An AUC score of 1 means that the model can perfectly predict all outcomes and a score of 0 means that the model predicts everything wrong. An AUC score of 0.5 means random guessing and AUC scores of 0.7–0.85 and higher are generally considered to be good to excellent in social sciences (e.g., Menaspa et al., 2010). We compared all models on their predictive performance via 12‐fold cross‐validation with AUC as the metric.

The first model was the baseline: a regularized linear model. Here, regularization was necessary because this study gathered relatively many variables compared to the number of observations. Without regularization, the model is likely to overfit in such situations. As regularization for the linear model, we choose Elastic Net which is a combination of Lasso and Ridge regression (for details, see Zou & Hastie, 2005) and fitted the model via MLJLinearModels.jl (Blaom et al., 2020). The strength of both regularizers was chosen automatically via hyperparameter tuning and 12‐fold cross‐validation. The second model was a decision tree, fitted via DecisionTree.jl (Sadeghi et al., 2022), and the third was a state‐of‐the‐art boosted random forest called XGBoost (Chen & Guestrin, 2016). The fourth model was a state‐of‐the‐art Stable and Interpretable Rule Sets (SIRUS) algorithm (Bénard et al., 2021; Huijzer et al., 2023). The SIRUS model is essentially also a random forest algorithm, but with a small modification such that it is more stable and, therefore, explainable. Note that contrary to more continuous models such as linear models, the rules fitted by SIRUS contain hard cutpoints (e.g., *if some variable* <20, *then A else B*).

Of these models, the XGBoost is the least explainable, while the other three models are all explainable. That is, the XGBoost cannot easily be interpreted due the complexity of the model. For the decision tree model, despite being explainable, it has the drawback of having a low stability since the split point at the root of the tree tends to vary wildly (for details about this phenomenon, see Molnar, 2022). The stability of the logistic regression is moderate since the model is highly sensitive to the choice of regularization parameters when using ridge, lasso, or both (Hastie et al., 2009). The stability of the XGBoost is high due to the large number of trees in the model which averages out fluctuations. Finally, the stability of SIRUS is generally high too since the algorithm was designed such that the structure of the random trees is more stable (Bénard et al., 2021). For more details about the analyses, see the code repository at osf.io (https://osf.io/c8hdy/?view_only=5d7765e9ffd543d98b51faae4802768a).

## 4 RESULTS

**FIGURE 1:**
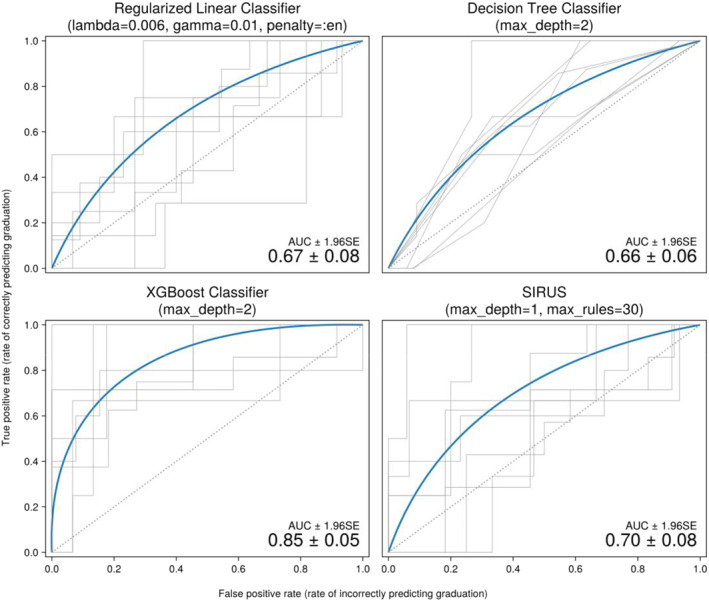
ROC Curves. The thick lines represent estimates of the average ROC curves over all folds. The smaller lines in gray display the variation on this estimate by showing the first eight folds in the 12‐fold cross‐validation. We show only eight folds because more folds made the plot very cluttered. The average area under the curve and 1.96 × standard error scores are shown in the bottom right. ROC, receiver operating characteristic.

The summary statistics of the variables are shown in Table A1 and correlations for all variables with graduation are shown in Figures A1 and A2. The Receiver Operating Characteristic (ROC) curves, average AUC scores, and standard errors are shown in Figure 1. To interpret these ROC curves, note that the diagonal line represents random guessing. Next, to create the lines, a model was fitted on one of the cross‐validation folds for each fold and used to predict data that the model had not seen during training. Then, note that a classification model can use different thresholds; the lower the threshold, the more likely an individual is classified as graduate. Finally, for each fold, the line is drawn by increasing the model threshold from 0 to 1 and comparing the model predictions to the true values. The AUC score is the averaged area under these curves.

The XGBoost model had the highest predictive performance, which was followed by the SIRUS model with a tree depth of 1 and at most 30 rules. Note that SIRUS with a tree depth of 2 would allow for more complex rules with two elements in the clause (e.g., *if X and Y*, *then A else B*) instead of only one clause (e.g., *if X*, *then A else B*). However, fitting a SIRUS model with a tree depth of two performed consistently worse, which indicated that the model overfitted the data. The logistic regression and the decision tree had slightly lower predictive performance.

**FIGURE 2:**
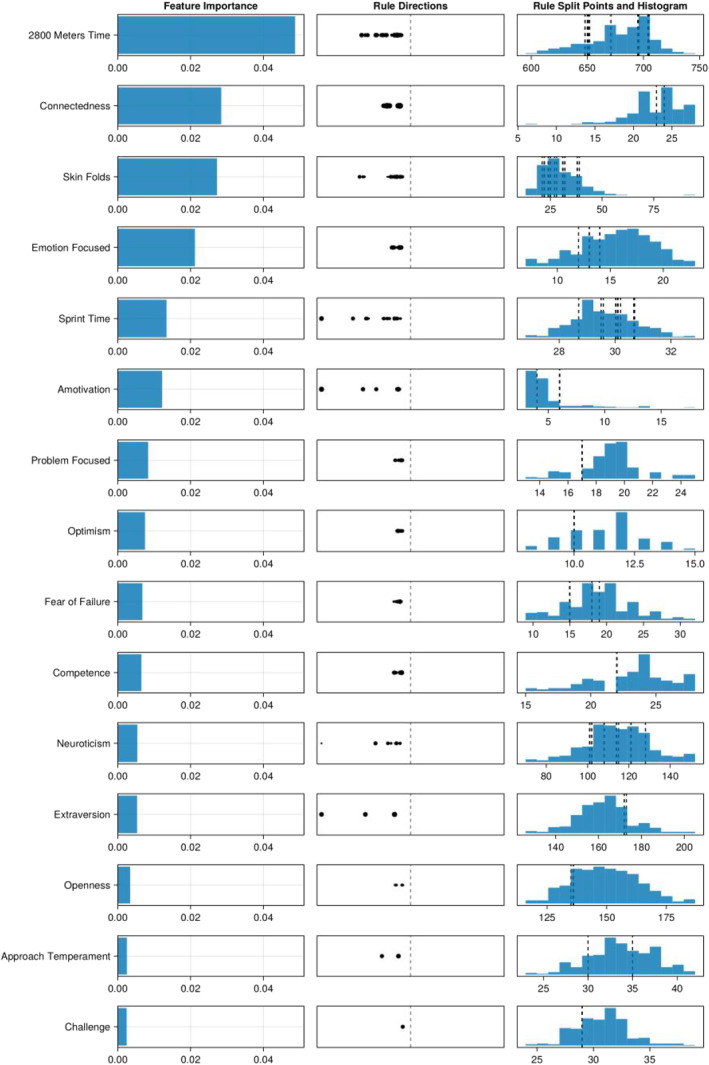
Rules used by the rule‐based classifier in different folds. This figure indicates the model uncertainty over different bootstrapped samples. The leftmost column shows the feature importance, the middle column shows the directions of the rules, and the rightmost column shows the split points of the rules and a histogram of the data. Specifically, the direction shows log (else‐scores/then‐scores). The sizes of the dots in the middle column indicate the weight that the rule has, so a bigger dot means that a rule plays a larger role in the prediction. These dots are sized in such a way that a doubling in weight means a doubling in surface size. Finally, the variables are ordered by the sum of the weights of the rules and only the first 15 are shown.

Altogether, while the XGBoost had a good predictive performance, the SIRUS model combined good predictive performance with strong stability and explainability (see Analysis section). We therefore decided to analyze the data further via this model. To do so, we have visualized the stability for different bootstrapped samples in Figure 2. Here, by bootstrapped samples, we mean that we took multiple random samples, via MLJ.jl (Blaom et al., 2020), of the data and fitted the model on each of these samples. The bootstrapping allowed us to visualize the uncertainty in the model which, in turn, aids model explanations.

To inspect the model, we go through one example feature in Figure 2. The figure shows that the 2800 m time had the most importance when summing the feature importances over the various bootstrapped samples. Next, we know that the rules in the SIRUS algorithm with a depth of 1 by default always point to “lower then”, for example, *if 2800 m time* < *650*, *then then‐score else else‐score* (Huijzer et al., 2023). If the *then‐score* is greater than the *else‐score*, then the model predicts that the individual who satisfies the rule is more likely to graduate. If the *then‐score* is smaller than *else‐score*, then the model predicts that the individual who satisfies the rule is more likely to drop out. The plotted rule directions show the direction of this *then‐score* and *else‐score* via log (else‐scores/then‐scores). Thus, from the plotted rule directions, we can see that the model found that a higher 2800 m time was associated with dropout. The exact locations of the split points (e.g., *if 2800 m time* < *650*) are shown in the right part of the plot and were different in the different bootstrapped samples. Most of the split points were at 650 s, and some were at 700 s. We plotted these split points on topof histograms of the data to show the distribution of the data.

When looking at all the predictions, the running time on the 2800 m was the most important with a clear cut‐off point for all folds at about 700 s. This means that, for all the folds, a higher running time was found to be associated with dropping out. Furthermore, a higher score on, in particular, need for connectedness or relatedness (a subscale of the need strength questionnaire) and skin folds were associated with dropping out.

### 4.1 Discussion

The purpose of the current study was to determine how well we could predict the dropout of special forces recruits while retaining model explainability and stability. To do so, we compared a linear, decision tree, XGBoost, and SIRUS classifier. Of the four models, the XGBoost had the best predictive performance. This is in line with earlier research that found that XGBoost is a powerful algorithm in a wide array of tasks ranging from predicting Tweet engagements (Anelli et al., 2020) to predicting injuries in competitive runners (Lövdal et al., 2021). However, XGBoost is less explainable than SIRUS. The difference between the two is that the SIRUS algorithm simplifies the model and then uses this model for both explanations and predictions. In contrast, model explainability methods typically use a simplified representation for explanations and the complex model for predictions. This difference between explanations and predictions could hide issues related to safety, fairness (e.g., biases), and reliability which is especially problematic in the context of selection, where wrong decisions can have a lasting impact on the individual. Next, the logistic regression, which is most familiar to sport and performance scientists, was explainable, but not very stable and performed slightly poorer than the SIRUS model. The general instability of the logistic model is an issue that has been described by Hastie et al. (2009). Furthermore, the decision tree is explainable but not stable (see Molnar, 2022). Together, the algorithm that displayed the best combination on all aspects was the SIRUS algorithm by achieving a good predictive performance and stability while remaining explainable.

The SIRUS algorithm appeared to be able to correctly deselect about 10%–20% of dropouts, that is, without sending recruits home who would have graduated, depending on the fold (see the top right of the SIRUS ROC in Figure 1). There is still a considerable amount of variance in the ROC curves, but at least 10% would already be a meaningful number in practice. Moreover, the accuracy of the prediction will most likely improve when fitting the model on the full dataset instead of cross‐validation folds and when gathering more data over time.

Since the SIRUS model performs relatively well and is explainable and stable, we can use our domain knowledge to estimate the generalizability of the model. With this in mind, the main takeaways from the current model are that candidates who take more than roughly 700 s on the 2800 m, score higher on need for connectedness, and have higher skin folds are more likely to drop out (see Figure 2).

Most of these variables are in accordance with earlier studies. For instance, a lower time for the 3‐mile run also predicted graduation in 800 U.S. special forces recruits (Farina et al., 2019). Furthermore, a lower fat percentage, as measured by the skin folds, was associated with physical fitness in 140 Finnish recruits (Mattila et al., 2007). Together, this adds theoretical confidence that the predictive model will generalize to new cohorts.

### 4.2 Limitations and future research

Although the psychological measurements were well organized and based on validated questionnaires, a limitation could be that participants faked their responses (e.g., Galić et al., 2012). To mitigate this in our study, we emphasized that data would be processed anonymously and that staff of the Special Forces unit could not access the data nor use it to make selection decisions, which has been shown to reduce the faking tendency (Kuncel & Borneman, 2007). Nevertheless, to make the transfer to real selection, the risk of faking should be accounted for. For future research, it would be interesting to investigate how selection decisions can be made on the data while new data keeps being added.

### 4.3 Conclusions and practical implications

In our attempt to predict dropout of special forces recruits by fitting machine learning models, SIRUS had a higher predictive performance than the linear classifier and decision tree, while being more explainable than the state‐of‐the‐art XGBoost classifier. In other words, SIRUS achieves a balance between predictive performance, explainability, and stability. This, together with its ease of use, makes it particularly suitable for many research problems in science, including selection in sports, and organizational and military contexts. This better understanding of the model may outperform the accuracy of black box models in the long run because it allows researchers to improve the model with their domain expertise and improve their domain expertise with the model. In turn, practitioners may use this to make data‐driven selection decisions.

To conclude, we would encourage scientists to use SIRUS, or similar stable rule‐based models. This is especially useful when working in fields, such as sports and military selection, where the number of variables often approaches the number of observations and where predictive performance, explainability, and stability are critical.

## CONFLICT OF INTEREST STATEMENT

The authors declare that they have no known competing financial interests or personal relationships that could have appeared to influence the work reported in this paper.

## Supporting information

Supporting Information S1
