# Supplementary material for: Predicting special forces dropout via explainable machine learning
Source: Eur J Sport Sci. 2024 Sep 24;24(11):1564–72. doi: 10.1002/ejsc.12162 (PMC11534633; doi:10.1002/ejsc.12162)
Supplement: Supplementary file 1 — Supporting Information S1 [file EJSC-24-1564-s001.pdf]

Appendix

The summary statistics are shown in Table A1. The correlations for the physical and psychological variables are respectively shown in Figure A1 and Figure A2.

Table A1

Summary Statistics

Feature	Minimum	Maximum	Median	Mean	SD
2800 Meters Time	595.0	746.0	680.5	678.51	27.39
Agreeableness	133.0	219.0	175.0	174.27	15.62
Amotivation	3.0	18.0	4.0	4.56	2.44
Approach Temperament	23.0	42.0	34.0	33.72	3.54
Autonomie	8.0	28.0	19.0	18.84	3.42
Autonomous Motivation	25.0	63.0	51.0	50.42	6.87
Avoidance Temperament	6.0	35.0	17.0	16.85	4.72
Bench Press	64.0	140.0	96.0	97.53	14.04
Challenge	24.0	39.0	31.0	31.21	2.42
Commitment	34.0	54.0	44.0	43.8	3.44
Competence	15.0	28.0	24.0	23.28	3.02
Confidence in Abilities	24.0	40.0	32.0	31.68	2.97
Connectedness	6.0	28.0	24.0	23.15	3.27
Conscientiousness	145.0	218.0	180.0	181.12	13.37
Controlled Motivation	6.0	38.0	17.0	17.69	6.12
Emotion Focused	7.0	23.0	16.0	15.57	3.17
Emotional Control	18.0	32.0	24.0	23.99	2.47
Extraversion	126.0	205.0	162.0	162.55	12.66
Fear of Failure	9.0	32.0	19.0	18.97	4.24
Grip Strength	72.8	159.0	114.25	114.07	16.89
Hex Bar	120.0	247.3	172.65	172.22	24.28
Interpersonal Confidence	14.0	30.0	22.0	22.14	2.56
Life Control	17.0	32.0	25.0	24.86	2.2
Matrix Reasoning	1.0	11.0	7.0	7.28	2.29
Mindset	6.0	30.0	20.0	19.38	4.6
Neuroticism	70.0	152.0	114.0	114.89	15.27
Openness	116.0	187.0	149.0	149.59	13.73
Optimism	8.0	15.0	12.0	11.29	1.53
Pessimism	3.0	12.0	7.0	6.89	1.86
Problem Focused	13.0	25.0	19.0	19.09	2.18
Pull-ups	6.0	24.0	14.0	13.94	3.43
Resilience	18.0	30.0	24.0	23.52	2.1
Seeking Support	9.0	25.0	18.0	17.99	2.64
Self-efficacy	620.0	1400.0	1050.0	1055.58	132.87
Skin Folds	13.0	95.0	28.0	29.65	9.28
Sprint Time	26.8	32.86	29.51	29.64	1.09
Sprint and Agility Time	7.06	11.89	10.0	10.02	0.51
Standing Long Jump	163.0	279.0	235.0	231.92	19.2
Three-dimensional Rotation	0.0	24.0	13.0	12.88	6.19

Figure A1

Correlations of the Physical Variables with Graduation

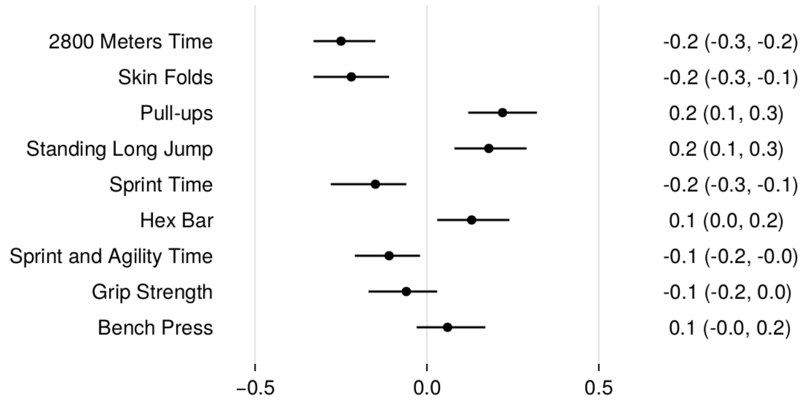

Note. The bars depict 95% confidence intervals as determined via Bootstrap.jl (Gehring et al., 2021).

Figure A2

Correlations of the Psychological Variables with Graduation

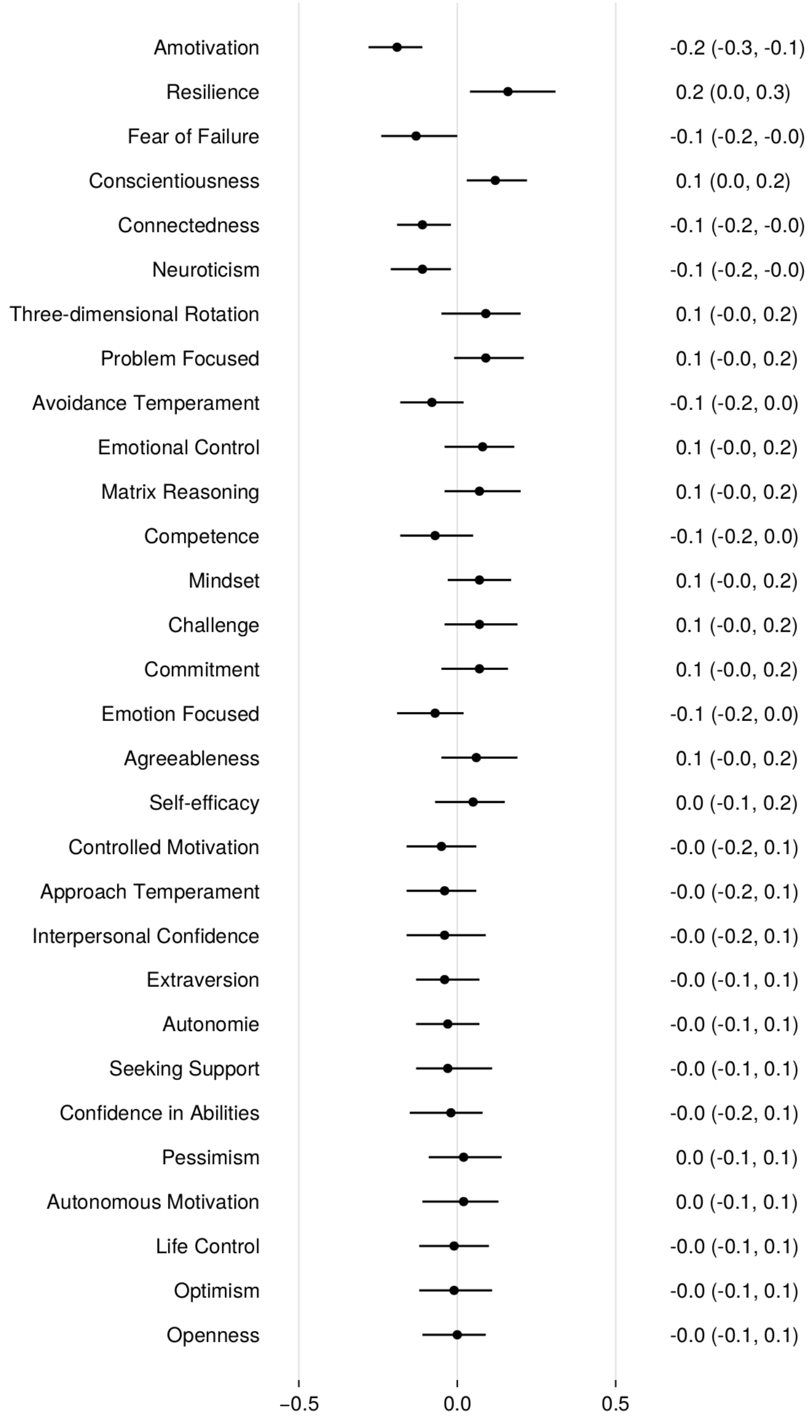

Note. The bars depict 95% confidence intervals as determined via Bootstrap.jl (Gehring et al., 2021).
